# Connecting the Missing Dots: ncRNAs as Critical Regulators of Therapeutic Susceptibility in Breast Cancer

**DOI:** 10.3390/cancers12092698

**Published:** 2020-09-21

**Authors:** Elena-Georgiana Dobre, Sorina Dinescu, Marieta Costache

**Affiliations:** 1 AMS Genetic Lab, 030882 Bucharest, Romania; dobregeorgiana_95@yahoo.com; 2 Department of Biochemistry and Molecular Biology, University of Bucharest, 050095 Bucharest, Romania; marieta.costache@bio.unibuc.ro; 3 The Research Institute of the University of Bucharest, 050095 Bucharest, Romania

**Keywords:** breast cancer, drug resistance, miRNAs, lncRNAs, biomarkers, therapeutic targets

## Abstract

**Simple Summary:**

Despite considerable improvements in diagnosis and treatment, drug resistance remains the main cause of death in BC. Multiple lines of evidence demonstrated that ncRNAs play a vital role in BC resistance. Here, we summarized the molecular mechanisms by which miRNAs and lncRNAs may impact the therapeutic response in BC, highlighting that these molecules can be further exploited as predictive biomarkers and therapeutic targets. By merging data from various studies, we concluded that several ncRNAs, such as miR-221, miR-222, miR-451, UCA1, and GAS5 are strong candidates for pharmacological interventions since they are involved in resistance to all forms of therapies in BC. Therefore, we believe that our review provides an important reservoir of molecules that may translate into clinically useful biomarkers, laying the ground for the adoption of ncRNAs within mainstream routine oncology clinical practice.

**Abstract:**

Whether acquired or de novo, drug resistance remains a significant hurdle in achieving therapeutic success in breast cancer (BC). Thus, there is an urge to find reliable biomarkers that will help in predicting the therapeutic response. Stable and easily accessible molecules such as microRNAs (miRNAs) and long non-coding RNAs (lncRNAs) are regarded as valuable prognostic biomarkers and therapeutic targets since they act as crucial regulators of the various mechanisms involved in BC drug resistance. Here, we reviewed the current literature on ncRNAs as mediators of resistance to systemic therapies in BC. Interestingly, upon integrating data results from individual studies, we concluded that miR-221, miR-222, miR-451, Urothelial Carcinoma Associated 1 (UCA1), and Growth arrest-specific 5 (GAS5) are strong candidates as prognostic biomarkers and therapeutic targets since they are regulating multiple drug resistance phenotypes in BC. However, further research around their clinical implications is needed to validate and integrate them into therapeutic applications. Therefore, we believe that our review may provide relevant evidence for the selection of novel therapeutic targets and prognostic biomarkers for BC and will serve as a foundation for future translational research in the field.

## 1. Introduction

Breast cancer (BC) is the most frequent malignancy diagnosed in women and the second leading cause of cancer-related deaths in females worldwide [1]. According to the most recent GLOBOCAN estimates, nearly 2.1 million women were diagnosed with BC worldwide in 2018, which resulted in 626,679 deaths [2]. Notably, late diagnosis [3] and the occurrence of drug resistance [4] are the main factors that account for the increased rate of fatality.

BC is a highly heterogeneous disease, clinically divided into three broad subgroups: hormone receptor-positive (that express receptors for estrogen and progesterone), human epidermal growth factor receptor 2 (HER2)-positive, and triple-negative breast cancer (TNBC), that lacks all of these receptors. Additional subtypes are now recognized, including luminal A, luminal B, HER2-enriched, claudin-low, basal-like, and normal breast-like, where the last three are part of TNBC. These subdivisions are different in terms of epidemiology, prognosis, and sensitivity to treatment and are very useful in guiding the therapeutic decisions [5,6]. In the last years, the combined use of endocrine, targeted, and cytotoxic agents has considerably improved the quality of life and survival in BC patients. However, a significant proportion of patients fails to respond to therapy due to the acquired or de novo drug resistance, which results in metastatic disease [7]. The recurrent disease usually spread to distant sites, such as the brain, bone, lung, and liver [8], and correlates with a dismal 5-year survival rate of 27% [9]. Enhanced drug efflux, alteration of drug targets (e.g., estrogen receptor (ER), human epidermal growth factor receptor 2 (HER2)), increased DNA damage repair (DDR), activated cancer stem cells (CSCs) and epithelial–mesenchymal transition (EMT), defective cell cycle control, apoptosis/survival pathways dysregulation, and the oncogenic signals within the tumor microenvironment (TME) have been extensively reviewed in association with the development of drug resistance in BC [10,11,12]. However, little is known about the processes driving the perturbation of these signaling pathways and the precise drug resistance-associated networks in BC [13].

**Figure 1:**
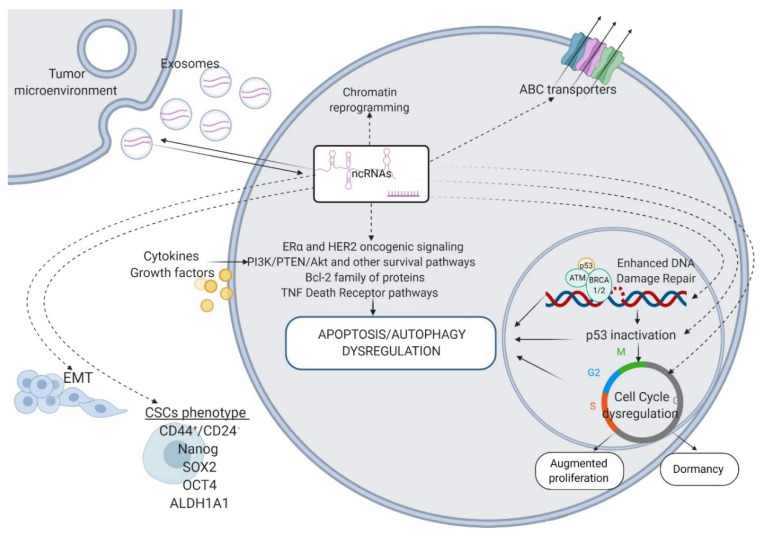
ncRNAs as key regulators of the mechanisms that render BC resistant to systemic therapies. ABC transporters—ATP-binding cassette transporters; Bcl-2—B-cell lymphoma 2; TNF—tumor necrosis factor; p53—tumor protein p53; ATM—Ataxia-Telangiectasia mutated; BRCA1/2—breast cancer 1/2; EMT—epithelial–mesenchymal transition; CSCs—cancer stem cells; CD44—cluster of differentiation 44; SOX2—SRY-Box Transcription Factor 2; OCT4—octamer binding transcription factor 4; ALDH1A1—aldehyde dehydrogenase 1 family member 1. Figure created with Biorender.com.

Non-coding RNAs (ncRNAs) are biologically active transcripts originating from the mammalian genome without protein-coding potential. These entities make up almost 97–98% of the human genome and are involved in various biological processes in health and disease, including the pathophysiology of BC. ncRNAs can be broadly divided into small non-coding RNAs (sncRNAs, <200 nucleotides) and long non-coding RNAs (lncRNAs, >200 nucleotides), based on their size [14]. There are different species of sncRNAs, including the notorious microRNAs (miRNAs), piwi-interacting RNAs (piRNAs), short interfering RNAs (siRNAs), small nuclear RNAs (snRNAs), and small nucleolar RNAs (snoRNAs) [15,16]. A novel addition to those sub-classes of ncRNAs are circular RNAs transcripts (circRNAs), generated through back-splicing of pre-mRNAs [17]. These molecules possess a unique circular conformation that endows them with enhanced stability compared to their linear RNA cognates. circRNAs are currently attracting considerable research attention as they are regulating multiple molecular mechanisms in health and disease, by which miRNA sponging is the most prominent [17,18,19]. miRNAs and lncRNAs are by far the best-studied ncRNAs. Recent data have demonstrated ncRNAs may play essential roles in the regulation of drug resistance in BC, by controlling autophagy, drug efflux, drug targets, chromatin state, cell cycle, DDR, apoptosis, angiogenesis, cellular stemness, and EMT (Figure 1) [20,21,22,23,24,25,26,27,28,29]. ncRNAs have also been found in exosomes, wrapped in a lipid bilayer, which protects them from degradation [30]. ncRNAs are shuttled in this form between different cell types residing within TME, thereby acting as critical regulators of intercellular signaling in BC. Furthermore, exosomal ncRNAs are entirely functional in the recipient cells, where they orchestrate dramatic changes that may help in spreading the resistance (Figure 1) [31]. miRNAs and lncRNAs act as oncogenes or tumor suppressors in cancers, and due to their functionalities, are currently explored as prognostic biomarkers and targets for therapeutic interventions in BC [32].

Here, we present a timely and detailed review of the latest research findings on the mechanisms that govern drug resistance acquisition in BC, focusing mainly on the regulatory roles of the aforementioned ncRNAs in these processes. Therefore, we consider that a comprehensive analysis of ncRNAs associated with drug resistance pathways is likely to provide new biomarkers for diagnosis and recurrence in BC patients, and potential targets for innovative therapeutic strategies.

## 2. Current Therapeutic Strategies and Associated Drug Resistance Profiles in BC

In BC clinical routine, the assessment of the immunohistochemical biomarkers (IHC), ER, progesterone receptor (PR), and HER2, altogether with the proliferation index (Ki67) is still the widely used protocol to determine the prognosis and eligibility for endocrine, targeted, or cytotoxic therapies [33].

**Figure 2:**
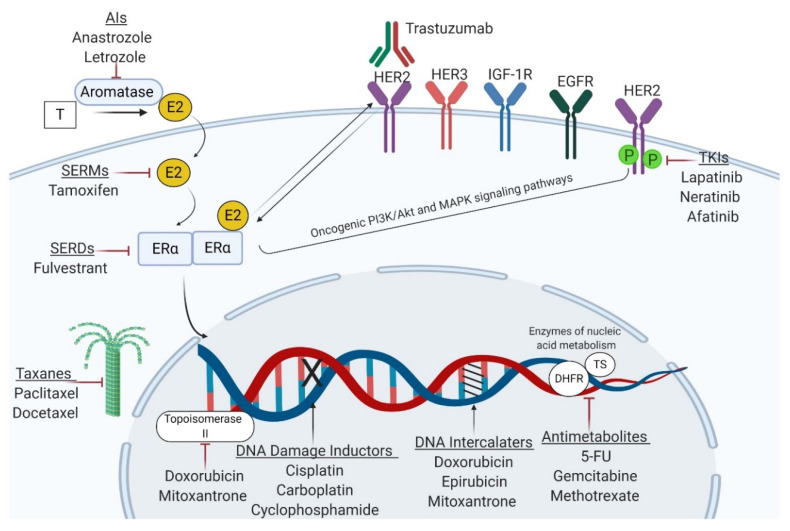
Mechanisms of action of the main classes of antineoplastic agents used in BC management. Endocrine therapies aim to interfere with ERα-associated signaling or decrease estrogen availability in the body, whereas trastuzumab and tyrosine kinase inhibitors (TKIs) are designed to block the HER2 receptor. Cytotoxic agents exert their anticancer effects through DNA damage (alkylating agents, platinum compounds), perturbation of nucleic acid synthesis (anthracyclines, taxanes, and mitoxantrone) or metabolism (antimetabolites). T—testosterone; E2—estradiol; ERα—estrogen receptor α; HER2—human epidermal growth factor receptor 2; IGF-1R—insulin-like growth factor 1 receptor; EGFR—epidermal growth factor receptor; TKIs—tyrosine kinase inhibitors; SERMs—selective estrogen receptor modulators; SERDs—selective estrogen receptor degraders; 5-FU—5-fluorouracil; DHFR—dihydrofolate reductase; TS—thymidylate synthetase. Figure created with Biorender.com.

ER is overexpressed in nearly 70% of BC patients, making them appropriate for endocrine therapies [34]. These therapies aim to disrupt the ERα-associated transcriptional activities (selective ER modulators (SERMs), such as tamoxifen) and orchestrate ER degradation (estrogen receptor down regulators (SERDs), such as fulvestrant). Additionally, it can lower the estrogens levels in the body by interfering with their synthesis (aromatase inhibitors (AI), such as anastrozole and letrozole) (Figure 2) [35]. For more than 40 years, tamoxifen (TAM) has been the gold standard in treating both premenopausal and postmenopausal women with ER-positive tumors. Its use has significantly decreased the mortality and disease relapse rates by 30% and 50%. However, 20–30% BC patients fail to respond to therapy, due to the intrinsic or extrinsic drug resistance, which often leads to metastatic disease and death [36]. The combined use of tamoxifen with AIs was shown to improve the therapeutic benefits in postmenopausal women, both in early and advanced stages [37]. However, the development of resistance to these first-line therapies remains a significant hurdle in BC management and calls for second-line therapies such as fulvestrant [38]. The dynamic interplay between ER signaling and important cell cycle regulators such as Cyclin-dependent kinase 4 and 6 (CDK4/6) has enabled the development of a new class of anticancer agents called CDK4/CDK6 inhibitors, which are extensively used in the treatment of metastasized ER-positive BC [39]. Their addition to traditional endocrine therapy, either in first-line or late-line setting has significantly improved the progression-free survival (PFS) and overall survival (OS) compared to endocrine therapy alone, in patients with metastatic ER-positive HER2-negative BC [40,41,42]; even so, their long-term efficiency seems to be also limited by the occurrence of drug resistance [43].

Similar to ER, HER2 is an important prognostic biomarker and therapeutic target in BC [44]. The HER2 receptor is amplified in 20–30% of BC cases and is associated with poor prognosis and aggressive behavior [45]. Several therapeutic formulations have been developed against HER2 receptor and its associated signaling, including monoclonal antibodies trastuzumab and pertuzumab, and tyrosine kinase inhibitors, TKIs (lapatinib, neratinib, afatinib) (Figure 2) [46]. Although the addition of trastuzumab to chemotherapeutic regimens has greatly improved the overall survival (OS) of HER2 patients, its efficacy is questionable, since a significant number of patients fail to respond to this therapeutic approach [47]. Both ER-positive and HER2-positive BC may be approached with chemotherapy, which remains the mainstay in triple-negative BC (TNBC) [48].

TNBC, notorious for its high proliferative rate and distant metastatic patterns is usually addressed with chemotherapeutics, such as anthracyclines (doxorubicin/adriamycin, epirubicin), taxanes (paclitaxel, docetaxel), platinum compounds (cisplatin, carboplatin), antimetabolites (methotrexate, 5-fluorouracil, gemcitabine), and alkylating agents (cyclophosphamide). Several chemotherapeutics such as doxorubicin and mitoxantrone may interfere with the catalytic activity of Topoisomerase II. In contrast, others such as alkylating compounds and platinum salts may induce DNA alkylation and subsequently, DNA damage. Furthermore, taxanes exert their antineoplastic effects through microtubules stabilization, whereas anthracyclines and mitoxantrone intercalate into DNA to prevent mitosis. Additionally, antimetabolites exert their cytotoxic activities via regulating key enzymes involved in nucleic acid metabolism to inhibit DNA synthesis and transcription (Figure 2) [35,47,49]. It is documented that systemic therapies have an overall efficiency of 90% in primary tumors and 50% in metastases. After a certain time, the tumor becomes refractory to medication, leading to recurrent disease [10].

Recent data have partially revealed the mechanisms associated with treatment failure to the main classes of antineoplastic agents used in BC. In luminal tumors, in which endocrine modulators target ER-associated signaling, ER loss, ER-tyrosine kinases receptors (RTKs) crosstalk that results in the activation of several oncogenic signaling pathways, along with cell cycle dysregulation, are the most common mechanisms that render tumors resistant to hormone therapies [12,50]. Similarly, loss of HER2, alternative signaling through other RTKs such as insulin-like grow factor 1 receptor (IGF-1R) and epidermal growth factor receptor (EGFR) and mutations in downstream signaling elements that lead to the subsequent activation of cell survival mechanisms may orchestrate the development of anti-HER2 drugs resistance in HER2-enriched BC [51]. Additionally, chemotherapy resistance is associated with elevated drug efflux, apoptosis and autophagy dysregulation, cell cycle arrest, enhanced repair of damaged DNA, and EMT induction [52]. Furthermore, there is strong evidence that ncRNAs, especially miRNAs and lncRNAs, are actively involved in regulating treatment sensitivity to almost all the therapeutic approaches available in BC [53,54,55,56,57,58,59]. The particular involvement of these ncRNAs in different BC-drug resistance-associated mechanisms will be outlined below.

## 3. miRNAs and BC Drug Resistance

miRNAs are a class of small non-coding molecules (20–22 nucleotides in length) that fine-tune gene expression by binding to 3 ‘untranslated regions (3′UTRs) of their target mRNAs, which results in translational repression or mRNA degradation [60]. The interaction of miRNA with a target transcript is mediated by a “seed sequence” of 2–8 nucleotides termed miRNA responsive element (MRE). A single transcript can harbor multiple MREs, being regulated by many miRNAs. At the same time, one miRNA can have MREs in the mRNA of many genes [61,62]. In cancers, tumor-suppressive miRNAs, such as let-7, miR-15/16, miR 200, miR-203, and miR-205 have more than expected target genes, while well-known cancer genes contain more than expected MREs [63]. The altered genomic distribution of miRNA target sites is thought to be responsible for the complexity of functional regulatory networks reported in tumors. Moreover, various ncRNAs, including lncRNAs, also interact with miRNAs, acting like the so-called “miRNAs sponges”. ncRNAs and mRNA share similar MREs and compete for a common pool of miRNAs, which results in modified mRNA translation and protein expression [62].

miRNAs biogenesis follows a multi-step process that includes the transcription, usually by RNA polymerase II, nuclear maturation and export, and formation of the effector miRNA-Argonaute complex termed as RNA-induced silencing complex (RISC) [30]. Nonetheless, certain miRNAs have been recently identified to be generated by alternative pathways that do not involve canonical components. These non-canonical miRNAs may have diverse origins and include Dicer-independent miRNAs, such as miR-451; miRtrons, which are DGCR8 Microprocessor Complex Subunit (DGCR8) and Drosha independent; simtrons, which are DGCR8, Dicer, Exportin-5, and Argonaute 2 (AGO2) independent, as well as snoRNA derived miRNAs and transfer RNA (tRNA) derived miRNAs [64,65]. As miRNAs regulate a plethora of cellular pathways, changes in biogenesis machinery may alter the expression and functionality of specific miRNAs, having important consequences in both health and disease [66,67,68].

miRNAs display increased evolutionary conservation and seem to be involved in the regulation of almost 60% of the protein-coding genes [69]. Interestingly, miRNAs have been found highly dysregulated in BC tissues compared to their matched normal counterparts [70,71]. Multiple lines of evidence suggest that they regulate almost all tumor biological properties, including the therapeutic response [28,59,72,73,74]. Notably, more than half of the miRNAs genes are located at fragile sites and cancer-associated genomic regions, suggesting a strong correlation between altered levels of miRNAs clusters and cancer biology [75]. However, the role of miRNAs in BC drug resistance to targeted, endocrine, and cytotoxic therapies will be outlined below.

### 3.1. miRNAs Involved in BC Resistance to Endocrine Therapies

Several studies investigated the correlation between estrogen resistance and miRNAs profiles in ER-positive BC cells in different experimental scenarios to provide a more accurate picture of the mechanisms underlying endocrine resistance in BC. For example, Ye and collaborators generated two tamoxifen (TAM)-resistant BC lines by two different administration methods: Michigan Cancer Foundation-7 C (MCF-7C) cell line via dose stepwise induction and MCF-7T cell line via temporal stepwise induction [76]. Therefore, the chemoresistance mechanisms displayed by MCF-7C would differ significantly from those activated in MCF-7T cells. Interestingly, the differential miRNAs profiles between TAM-sensitive (MCF-7) and TAM-resistant (MCF-7C, MCF-7T) BC cell lines analyzed by high-throughput RNA sequencing displayed differences in the levels of 118 miRNAs between MCF-7 and MCF-7C lines (*p* < 0.05), respectively, 42 miRNAs between MCF-7T and MCF-7 cells (*p* < 0.05). They also found that 75 miRNAs were increased, and 50 miRNAs were decreased significantly in MCF-7C versus MCF-7T cells. Additionally, Ye and colleagues pointed out that miR-21, miR-146a, miR-148a, miR-34a, and miR-27a may play essential roles in the acquisition of the TAM-resistant phenotype and become potential targets for TAM-resistant BC [76]. In parallel, Zhou and his team analyzed miRNAs expression profiles in fulvestrant and TAM-resistant tumors and found that miR-4532, miR-486-5p, miR-138, miR-1228, and miR-3178 may be promising candidates for reversing endocrine resistance in BC. They also showed that miR-3188, miR-21, miR-149 might be associated with fulvestrant resistance, while miR-342 and miR-1226 may promote TAM resistance [77]. Therefore, comparative analysis of these profiles may lead to a better understanding of the mechanisms underlying anti-estrogen resistance and may encourage the development of new targeted therapies that may be administered in conjunction with TAM or fulvestrant in BC.

Other studies have examined the involvement of individual miRNAs in resistance to endocrine therapies (Table 1). One of the pioneering studies in the field found that low levels of miR-342 may serve as a potential signature of TAM resistance in BC, being positively associated with BC recurrence and metastasis. miR-342 acts as a tumor suppressor and can sensitize ER-positive tumors to endocrine therapy by regulating genes involved in cell death pathways, such as gem nuclear organelle associated protein 4 (GEMIN4) and bone morphogenetic protein 7 (BMP7) [78]. miR-320 is another tamoxifen sensitizer as this miRNA target cAMP-regulated phosphoprotein (ARPP-19) and estrogen-related receptor gamma (ERRγ), along with their downstream effectors c-Myc and Cyclin D1 [79]. Additionally, miR-451a was shown to restore TAM-MCF-7 sensitivity to tamoxifen by targeting 14-3-3ζ, a crucial factor that binds and protects proteins with critical roles in apoptosis and cell survival, including EGFR, HER2, β-catenin, and proto-oncogene serine/threonine-protein kinase (RAF-1). miR-451 induction inhibited EGFR, HER2, and mitogen-activated protein kinase (MAPK) signaling and enhanced apoptosis in TAM-resistant BC lines, suggesting that miR-451 may have therapeutic potential for ER-positive BC [80]. Additionally, miR-15a/16 may increase tamoxifen efficiency in BC via Cyclin E1 and B-cell lymphoma 2 (Bcl-2) modulation [81].

Among miRNAs with oncogenic functions in endocrine resistance is miR-21. This miRNA is involved in the development of resistance to almost all therapeutic approaches available in BC, including tamoxifen, fulvestrant, trastuzumab, and gemcitabine [82,83,84]. miR-21 is documented to be overexpressed in ER-positive BC lines and promote tamoxifen and fulvestrant resistance by targeting phosphatase and tensin homolog (PTEN) of the phosphatidylinositol 3-kinase/protein kinase B (PI3K/Akt) signaling pathway in BC [82]. Another study revealed that miR-221/222 released from TAM-MCF-7 could enter wild-type MCF-7 cells, endowing them with tamoxifen and fulvestrant refractoriness via p27 and ERα modulation, thereby enabling tumor growth in an ER-independent manner. One step further, pathway analysis elucidated that miR-221/222 transfer was correlated with the hyperactivation of several oncogenic pathways such as p53, transforming growth factor-beta (TGF-β), MAPK, Notch, ErbB, Janus kinase/signal transducers, and activators of transcription (Jak/STAT) signaling [55,85]. As an added complication, cancer-associated fibroblasts (CAFs) may also release miR-221-enriched exosomes to propagate fulvestrant resistance into tumor stroma in an interleukin (IL-6)-dependent manner [86]. Another oncogenic miRNA is miR-155, which was documented to promote tamoxifen resistance via Suppressor of Cytokine Signaling 6 (SOCS6) targeting the STAT3 pathway [87].

### 3.2. miRNAs Involved in BC Resistance to Targeted Therapies

Several studies have revealed a mechanistic link between miRNAs dysregulation and trastuzumab resistance in BC. For example, Yang and collaborators suggested that 48 miRNAs are upregulated, and 105 miRNAs are downregulated in trastuzumab-resistant JIMT-1 cells compared to their sensitive counterparts SKBR3. The cellular levels of seven miRNAs were further confirmed by qRT-PCR, and the Kyoto Encyclopedia of Genes and Genomes (KEGG) analysis revealed that they are mainly involved in the modulation of the PI3K/AKT pathway. They further used these seven miRNAs to construct a serum miRNAs signature that predicts trastuzumab response and found that miR-135b, miR-200b, and miR-29a are upregulated, whereas miR-224 is significantly downregulated in the plasma of the HER-2 enriched BC patients that do not benefit from trastuzumab [96]. Li and his team proposed another plasma miRNAs signature to distinguish between sensitive and non-sensitive trastuzumab patients. They suggested that miR-940 downregulation and miR-451a, miR-16-5p, and miR-17-3p overexpression are associated with increased trastuzumab efficiency [97]. Of particular importance, Di Cosimo and collaborators have recently reported that an increase in miR-148a-3p levels during trastuzumab administration is associated with an optimistic prognosis; additionally, the pathologic complete response (pCR) improved, even more, when miR-140-5p levels were concomitantly increasing [98].

However, other studies focused on the involvement of a particular miRNA in BC targeted-therapy resistance. Epigenetic silencing of miR-375 plays a crucial role in the development of trastuzumab resistance. It is associated with increased levels of its target IGF-1R, which may function as an alternative growth factor receptor in patients subjected to trastuzumab [99]. Another miRNA with a high affinity for IGF-1R is miR-630, found highly downregulated in HER2 metastatic and TKIs-resistant BC patients. Since miR-630 overexpression may restore sensitivity to HER2-targeted agents by attenuating tumor cell aggressiveness and motility, miR-630 can be regarded as a putative diagnostic and prognostic biomarker in lapatinib resistance but also as a therapeutic alternative in removing TKIs resistance in BC [100]. Another miRNA involved in trastuzumab resistance is miR-210, which regulates the transcription factor E2F3 and the DNA repair enzyme RAD52 to support tumor growth and dissemination upon previous treatment exposure [101,102]. miR-21 and miR-221 are other two oncogenic miRNAs that promote trastuzumab resistance via PTEN silencing and subsequent PI3K/Akt signaling hyperactivation [103,104].

In contrast, miR-16 acts as a tumor suppressor and can restore trastuzumab and lapatinib sensitivity by suppressing Cyclin J (CCNJ) and Far Upstream Element Binding Protein 1 (FUBP1) in resistant BC [105]. The exosomal transfer of miR-567 was recently reported to reverse trastuzumab resistance in BC via Autophagy related 5 (ATG5) inhibition; thereby, its therapeutic addition may considerably improve and enhance patients’ responsiveness to this drug [106]. Other miRNAs with oncogenic or tumor suppressor functions in anti-HER2 drug resistance can be found in Table 2.

Of particular importance, miR-205 has been proved to play antithetical roles in regulating the response to targeted therapies in BC. miR-205 has been consistently reported downregulated in mammary carcinomas versus normal breast tissues. It emerged as an onco-suppressive miRNA due to its ability to suppress several notorious oncogenes, such as ErbB3 and Zinc Finger E-Box Binding Homeobox 1 (Zeb1). Cataldo and his team confirmed the tumor-suppressive function of miR-205 in HER2-positive BC cell lines, as its ectopic expression improved trastuzumab effectiveness in vitro through the impairment of Akt signaling pathway [26]. Surprisingly, De Cola et al. found that miR-205 was upregulated in HER2-positive patients derived-breast cancer stem cells, and its increased levels promoted targeted therapy resistance via downregulating ERBB2 and EGFR in a p63-dependent manner [24]. The different functions exerted by miR-205 in this clinical setting suggest that developing an appropriate strategy in removing drug resistance is not as straightforward as some researchers postulated in their studies.

### 3.3. miRNAs Involved in BC Chemoresistance

Several miRNAs have been documented to act on ATP-binding cassette (ABC) transporters, which may confer chemoresistance in cancers by pumping the drugs out of the cell, thereby decreasing their intracellular concentration. As these membrane transporter proteins may act on various substrates, they have been inherently associated with the acquisition of multidrug resistance (MDR) phenotype in BC [109]. Multidrug resistance protein 1/P-glycoprotein (MRP1/P-gp), the archetypal representative of this family, is actively involved in BC cancer refractoriness to a variety of compounds, including anthracyclines, platinum agents, and taxanes [110]. P-gp overexpression resulting from decreased miR-451 levels is associated with anthracycline resistance in MCF-7 cells [111]. Interestingly, miR-302a/b/c/d has been shown to reverse the MDR phenotype in BC cells by indirectly repressing P-gp following Mitogen-activated protein kinase kinase kinase 1 (MEKK1) targeting in the extracellular-signal-regulated kinase (ERK) pathway [25]. MRP pumps, which act on similar substrates as P-gp, are also subject to miRNA regulatory action. For instance, loss of miR-7 and miR-345 have been documented to contribute to MRP1-mediated cisplatin resistance in BC, whereas the reinforcement of these two miRNAs sensitized ER-positive BC cell lines to platinum agents [53]. Additionally, miR-489 increased BC therapeutic response to doxorubicin and cisplatin by reducing MRP-2 levels, a recurrent transporter found in platinum-resistant cells [53]. Other miRNAs associated with chemoresistance, including ABC-transporter mediated resistance may be found in Table 3.

Many chemotherapeutic agents used in BC, such as platinum compounds and alkylating agents exert their antineoplastic effects through DNA damage. Indeed, several pathways are activated in response to genotoxic stress, including cell cycle arrest and DDR [33]. Of particular interest, certain miRNAs that act on genes involved in cell cycle control and DNA repair have been proposed to serve as potential biomarkers for prognosis and therapeutic response in BC patients. For example, miR-34a may downregulate Bcl-2 and attenuate Cyclin-D1-G1-induced arrest to promote docetaxel resistance in mammary carcinomas [54]. Additionally, low levels of miR-302b have been shown to promote cisplatin resistance via E2F1 and DDR upregulation in BC [112]. Interestingly, the miR-449 family has been shown to regulate several genes involved in cell proliferation, such as E2F1, E2F3, and Cyclin-dependent kinase 2 (CDK2) to enhance doxorubicin cytotoxicity in TNBC cell lines [113]. Moreover, miR-638 and miR-218 can target Breast Cancer 1 Gene (BRCA1) and interfere with tumor cells’ abilities to repair cisplatin-induced DNA damage, increasing the efficiency of platinum salts in BC [114,115].

Apoptosis avoidance is also a ubiquitous phenomenon in non-responsive breast tumors, and many authors have potentiated a mechanistic link between miRNAs and apoptosis deregulation in BC (Table 3). Overexpression of miR-29a and miR-222 was associated with BC anthracycline and taxane resistance, through PTEN downregulation and the subsequent activation of the Akt/mTOR signaling pathway [116]. Low levels of miR-205 were also reported in BC resistant to docetaxol, doxorubicin, and cyclophosphamide regimens. Notably, miR-205 induction was found to downregulate vascular endothelial growth factor A (VEGFA), and fibroblast grow factor-2 (FGF2), which results in impaired PI3K/Akt signaling and increased apoptosis upon chemotherapy [27]. miR-100 is also downregulated in drug-resistant BC, and its forced expression was proved to sensitize tumors to paclitaxel by targeting mTOR and its associated signaling [117]. miR-542-3p is another biomarker of paclitaxel sensitivity in BC and has been documented to act on the antiapoptotic protein survivin leading to attenuated HER3/PI3K/Akt signaling, which enhanced paclitaxel cytotoxic activity in HER2-positive tumors [118]. miRNAs also exert their pro- or antiapoptotic activities downstream of the Akt pathway. For example, Bcl-2 overexpression due to low levels of miR-451 [119], miR-181a [120], miR-424/322, and miR-503 [121] can counteract chemotherapy induced apoptosis resulting in limited therapeutic efficiency, disease relapse, and metastasis. Additionally, miR-221 [122] and miR-944 [123] were found to prevent BC from undergoing apoptosis and instigate cisplatin resistance through direct repression of Bcl-2-like protein 11 (Bim) and BCL2/adenovirus E1B 19 kDa protein-interacting protein 3 (Bnip-3), respectively. In contrast, miR-31 was shown to render BC more vulnerable to medication via Bcl-2 downregulation following Protein Kinase C Epsilon (PKCε) targeting in the STAT3 pathway [124]. Interestingly, miRNAs can also act on molecules involved in the final stages of apoptosis, such as caspases to regulate the apoptotic response in BC. One such miRNA is let-7, which has been confirmed to attenuate doxorubicin-induced apoptosis through translational repression of effector caspase-3, a master regulator of programmed cell death [125].

miRNAs can also modulate the therapeutic response via regulating autophagy (Table 3). A recent study revealed that isoliquiritigenin, a natural flavonoid has the property to suppress miR-25a and induce autophagic cell death in BC drug-resistant tumors both in vivo and in vitro. Further analysis potentiated that miR-25a inhibition leads to the amplification of its target Unc-51 like autophagy activating kinase 1 (ULK), which may restore doxorubicin sensitivity through ABCG2 degradation via the autophagy-lysosome axis [126]. Similarly, miR-489 can increase doxorubicin efficiency in BC by blocking autophagy as an alternative survival mechanism under stressful conditions [28]. Additional studies revealed ULK1 and lysosomal transmembrane protein 4 beta (LAPTM4B) as direct targets of miR-489 [28]. Currently, the mechanistic link between miRNAs and autophagy in BC chemoresistance is extensively investigated, as it may reveal new therapeutic strategies for approaching BC resistant tumors.

The presence of breast cancer stem cells (BCSCs), a minor population from the tumor bulk that displays stem cell properties, is another crucial determinant of chemoresistance in BC. BCSCs refractoriness to conventional therapies may be partially attributed to their quiescent phenotype which is inappropriately addressed with antiproliferative chemotherapy; however, in recent years, a strong relationship between miRNAs and the regulation of CSCs features including tumorigenic potential, pluripotency, and EMT with severe implications in therapeutic response has been described [33]. Thereby, understanding the miRNAs involved in CSCs regulation may reveal new therapeutic approaches to circumvent the chemoresistance hurdle in BC. In doxorubicin- and cisplatin-resistant TNBC cells, miR-137 is overexpressed and leads to increased levels of its target gene Follistatin-related protein 1 (FSTL1), which seems to be involved in stemness maintenance and chemoresistance via Wnt/β-catenin signaling modulation [29]. miR-140-5p may also act on the Wnt/β-catenin pathway to suppress the self-renewal potential and tumorigenicity of BCSCs while restoring the sensitivity of tumors to anthracyclines [127]. Furthermore, low levels of miR-155 sensitized tumors to doxorubicinol by modulating several markers associated with BCSCs phenotype, including CD44, CD90 cell surface biomarkers, and ABCG2 protein transporter [128]. The overexpression of drug transporter proteins and pro-apoptotic proteins in the mitochondrial pathway is another common mechanism exploited by CSCs to prevent chemotherapy-induced apoptosis [33]. For instance, miR-519d was found profoundly downregulated in BCSCs, and its forced induction restored cisplatin sensitivity via pro-apoptotic Bcl-2 family protein myeloid cell leukemia 1 (MCL-1) modulation (Table 3) [129].

EMT, the process by which non-motile epithelial cells from the primary site acquire migratory and invasive potential and become mesenchymal cells, is also documented to provide cells with CSCs properties and affect the patient’s prognosis [130]. miR-21 can orchestrate EMT and hypoxia-inducible factor 1-alpha (HIF-1α) activation, increasing the invasiveness and adaptability of third-sphere, forming BCSCs-like to hypoxic conditions [21]. Notably, low levels of miR-30c may promote BC chemoresistance via overexpression of EMT-related cytokines Twinfilin 1 (TWF1) and IL-11 [131]. Moreover, miR-155 delivered by exosomes augmented EMT and conferred chemoresistance to recipient BC cells, by regulating at least five genes: adenomatous polyposis coli (APC), Hydroxysteroid 17-Beta Dehydrogenase 12 (HSD17B12), MYC, Mothers against decapentaplegic homolog 1 (SMAD1), and SMAD3 [132]. Additionally, miR-155 has been described downregulate CCAAT/enhancer-binding protein beta (C/EBP-β), which resulted in TGF-β-induced EMT, metastasis, and indeed, chemoresistance [74]. miR-200c, highly underexpressed in BC and associated with aggressive behavior, has been indicated to act on B lymphoma Mo-MLV insertion region 1 homolog (BMI), leading to EMT activation and increased tumorigenicity [133]. Interestingly, the induction of miR-200c in claudin-low tumors, which primarily express EMT and CSCs features significantly reduced chemoresistance via colony-forming capacity downregulation and EMT disruption, providing a promising therapeutic strategy for highly aggressive TNBC tumors [134]. All this information offers valuable insights into the impact of miRNome on the properties of CSCs and can be exploited for forecasting or developing theranostic strategies to combat the drug-resistance phenotype in BC.

Another factor influencing the sensitivity of cancers to chemotherapy results from their interaction with the local microenvironment [33]. Mainly, BCSCs can transfer exosomal miRNAs to neighboring sensitive cells, and it may have a tremendous impact on metastasis, tumor growth, and therapeutic response. For example, miR-222 enriched exosomes of doxorubicin-resistant MCF-7 cells transferred the chemoresistance to sensitive cells through negative regulation of PTEN [135]. Similarly, TNBC cells derived exosomal miR-1246 were shown to enhance Human Mammary Epithelial (HMLE) cell line proliferation, migration, and multidrug resistance via Cyclin G2 (CCNG2) downregulation [136]. Tumor-associated macrophages (TAM) can also take up miR-770 from TNBC tumor cells, and further analysis revealed that miR-770 negatively regulates gene coding for Stathmin 1 (STMN1). STMN1 is also known as metablastin and oncoprotein 18, triggering M1 polarization of macrophages and EMT disruption, which further results in increased sensibility to doxorubicin [137]. BC cell dormancy in the bone marrow (BM) is also associated with chemoresistance in BC, and exosomal miRNAs transfer appears to play crucial roles in this process [138,139]. For example, in a coculture system, BM-mesenchymal stem cells (MSCs) can deliver miR-23b to BC cells, triggering quiescence by suppressing the target gene myristoylated alanine-rich C kinase substrates (MARCKS), involved in cell cycle progression and motility [140]. Similarly, MSCs-derived miR-222/miR-223 can induce dormancy and chemoresistance in neighboring tumor cells, but the mechanisms involved remain to be elucidated [141].

## 4. lncRNAs in BC Drug Resistance

Recently, another class of ncRNAs, the lncRNAs have gained tremendous attention because of their multilayered regulatory activities. Unlike endogenous miRNAs, lncRNAs are RNA transcripts that extend over 200 nucleotides, abundant in the human genome and with high tissue specificity [188,189]. Recent evidence highlighted that core promoter sequences play key roles in regulating transcript tissue specificity. Briefly, increased tissue specificity is associated with less transcription factor (TF) motifs at the core promoter [190,191]. Interestingly, the tissue/stage/subtype-specific expression of lncRNAs positions them as promising prognostic and diagnostic biomarkers, as well as therapeutic targets for a variety of pathologies [192].

Based on their localization within the protein-coding genes, lncRNAs can be classified into five subtypes: sense lncRNAs, antisense lncRNAs, intergenic or intronic transcripts, and bidirectional lncRNAs [193]. lncRNAs have a complex structure (secondary or tertiary) that allows them to interact with various molecules such as DNA, mRNA, other ncRNAs (e.g., miRNAs), and proteins. This may be why lncRNAs are actively involved in gene regulation, modulating vital cellular processes such as chromatin reprogramming, transcription, post-transcriptional processing, and translation [20,188,194]. However, based on their functionalities, they have been divided into four archetypes: (1) signals, (2) decoys, (3) guides, and (4) ropes [195]. lncRNA biogenesis is similar to that of miRNAs, being transcribed by RNA polymerase II. lncRNAs are spliced, capped, and polyadenylated and usually transported to the cytoplasm. However, lncRNAs reside and operate both in the nucleus and in the cytoplasmic compartment. Nuclear lncRNAs may exert their cis- and trans-regulatory activities by regulating gene expression at local or distal sites. In contrast, cytosolic lncRNAs can act as competitive endogenous RNAs (ceRNAs), sequestrating miRNAs, and subsequently influence the expression of different miRNA-target genes [196].

Currently, over 30,000 transcripts have been identified and annotated within the human genome, and their number is expected to increase over time [197]. Due to their heterogeneity and abundance, lncRNAs are poorly characterized, compared to sncRNas. However, what was once considered “transcriptional noise” now has well-defined roles in different cell types and organisms. Studies have shown that lncRNAs strictly regulate physiological processes and that their expression profiles are highly dysregulated in cancers, including BC [198,199,200]. The deregulation of the transcriptomic landscape may have severe implications for metastasis, tumor growth, and, indeed, response to medication [201,202,203]. Herein, we will focus mainly on the lncRNAs involved in modulating susceptibility to conventional therapies in BC.

### 4.1. lncRNAs and BC Resistance to Endocrine Therapy

Regarding the involvement of lncRNAs in resistance to endocrine therapies, most of the molecules identified so far have been associated with tamoxifen resistance, as it is the gold standard in the treatment of ER-positive BC.

A recent article proposed an 11 lncRNAs-based tool for estimating the risk of relapse among ER-positive BC patients addressed with tamoxifen. The increased expression of RP11.259N19.1, KB.1460A1.5, and PP14571, along with low levels of PTEN-induced kinase 1 (PINK1) Antisense RNA (PINK1.AS), KLF3 antisense RNA 1 (KLF3.AS1), Long Intergenic Non-Protein Coding RNA 00,339 (LINC00339), LINC00472, Retinitis pigmentosa-11.351I21.11 (RP11.351I21.11), PKD1P6.NPIPP1, PDCD4 Antisense RNA 1 (PDCD4.AS1), KLF3 antisense RNA 1 (KLF3.AS1), PP14571, and RP11.69E11.4 were associated with an increased risk of recurrence in tamoxifen-resistant BC, and the 3/5 year survival rates further confirmed this in clinical cohorts. However, no significant association was reported between the expression levels of these lncRNAs and clinic-pathological factors. Interestingly, pathway analysis performed on the 11 tamoxifen resistance-related lncRNAs highlighted Akt and Wnt signaling as the most prominent pathways in this clinical setting [204]. Evidence regarding lncRNAs involvement in endocrine resistance also comes from individual studies, and the scientific efforts made so far have resulted in the identification of at least five oncogenic lncRNAs (HOTAIR, BCAR4, UCA1, CCAT2, ROR) and a single suppressor tumor, GAS5 in the clinical setting of BC endocrine therapy resistance (Table 4).

HOX antisense antigenic RNA (HOTAIR) is an oncogenic lncRNA able to recruit Polycomb Repressive Complex 2 (PRC2) and Lysine-specific demethylase 1 (LSD1) complex to promote carcinogenesis and metastasis in many cancers, including BC [20,194]. Enhanced expression of lncRNA HOTAIR was shown to support tamoxifen resistance in BC through direct interaction with ERα, which resulted in the amplification of ER-associated transcriptional programs even in the absence of estrogens [205]. The newly discovered HOX antisense intergenic RNA myeloid 1 (HOTAIRM1) was also associated with tamoxifen resistance via HOXA genes modulation [206]. HOTAIRM1 and HOXA1 were found overexpressed in tamoxifen-resistant tumors, and their silencing led to increased therapeutic response. Subsequent studies have shown that HOTAIRM1 is capable of interacting with the Enhancer of zest homolog 2 (EZH2) of PRC2 and prevent the deposition of H3K27me3 marks at the HOXA1 promoter, thereby increasing HOXA1 levels in tamoxifen-resistant BC cells. Therefore, HOTAIRM1 and HOXA1 may serve as promising targets in ER-positive patients who have acquired tamoxifen resistance [206].

Overexpression of Breast Cancer Anti-Estrogen Resistance 4 (BCAR4), associated with rapid disease progression and poor prognosis, has also been proposed as a signature of tamoxifen resistance in BC. It was found that BCAR4 stimulates the phosphorylation of v-erb-b2 erythroblastic leukemia viral oncogene homologs (ERBB2) and ERBB3, together with their downstream regulators AKT and extracellular signal-regulated kinase 1/2 (ERK1/2) [207]. siRNA-mediated reduction of ERBB2 and ERBB3 blocked BCAR4-driven proliferation, confirming the crucial role of this lncRNA in tamoxifen resistance in BC. Interestingly, the addition of lapatinib to antiestrogenic regimens has proven to be an effective strategy in BCAR4-driven ER-positive breast tumors, since lapatinib can disrupt ERBB2 phosphorylation and the stimulation of its downstream mediators AKT, FAK, SHC, STAT5, and STAT6. However, a group of BCAR4-positive/ERBB2-low-expressing BC patients eligible for tamoxifen and lapatinib regimens has been shown not to benefit entirely from this therapy, suggesting that BCAR4 silencing may be a useful therapeutic approach just in certain subgroups of resistant BC [208]. Colon Cancer Associated Transcript 2 (CCAT2), another oncogenic lncRNA that controls tumor growth and dissemination by modulating Wnt and TGF-β signaling in BC, has been found in increased levels in tamoxifen-resistant BC [209,210]. CCAT2-mediated drug resistance was further documented to be supported by ERK/MAPK signaling, and the silencing of either ERK/MAPK and CCAT2 resulted in increased apoptosis and necrosis in tamoxifen-resistant BC [211]. lncRNA regulator of reprogramming (ROR) is also associated with enhanced proliferation, invasion, and hormone therapy resistance in BC via regulation of the miR-205-5p/ZEB1/ZEB2 axis [202]. Alternatively, lncRNA ROR was shown to regulate MAPK/ERK signaling to confer estrogen-independent and cell growth in BC [212].

lncRNA Urothelial Carcinoma Associated 1 (UCA1) is also overexpressed in tamoxifen-resistant BC cell lines and its silencing results in G2/M arrest, enhanced apoptosis, and increased sensitivity to anti-estrogens. It was further shown that UCA1 binds to the homologous enhancer of zeste 2 (EZH2) of the p21 promoter and downregulates the p21 gene; however, further studies have shown that UCA1-mediated tamoxifen resistance relies on the PI3K/Akt pathway [213]. Beside AKT/mTOR signaling, UCA1 was documented to regulate the Wnt/β-catenin pathway in hormonotherapy-resistant BC [214,215]. On another mechanism, UCA1 acts as a molecular sponge for miR-18 and promotes BC drug resistance by attenuating the inhibitory effects of miR-18 on HIF1α expression [216]. Last but not least, the transfer of UCA1-enriched exosomes from tamoxifen-resistant to tamoxifen-sensitive BC cells resulted in enhanced proliferation and decreased levels of cleaved caspase-3 and apoptosis in the recipient cells. It was further confirmed that exosomes with impaired UCA1 transferred to MCF-7 cells could not render tumors refractory to drugs, suggesting that UCA1 knockdown may be an effective strategy to overcome tamoxifen resistance in BC [217].

Among lncRNAs with tumor suppressor function is Growth arrest-specific 5 (GAS5). GAS5 was found significantly downregulated in mammary carcinomas, and its status was positively associated with advanced TNM stages, poor prognosis, and tamoxifen resistance. Experimental induction of GAS5 sensitized MCF-7/R cells to tamoxifen via its ceRNA interaction with miR-222, a negative regulator of PTEN, which resulted in PI3K/Akt signaling inhibition [218].

### 4.2. lncRNAs and Resistance to Anti-HER2 Drugs

The roles of lncRNAs in the clinical setting of anti-HER2 drug resistance are also beginning to be elucidated. Genome-wide profiling of differentially expressed lncRNAs in trastuzumab-resistant tumors and healthy tissues revealed 30 significantly deregulated lncRNAs and long noncoding RNA activated by TGF-β (lncRNA ATB) was by far the most overexpressed. ATB has been shown to promote trastuzumab resistance and metastasis in BC through direct sequestration of miR-200c, leading to ZEB1 and Zinc finger protein 17 (ZNF217) overexpression and EMT [56]. ZNF217 supports TGF-β signaling through TGF-β2 and TGF-β3 transcriptional activation; interestingly, TGF-β signaling hyperactivation results in ZEB1 overexpression, outlining a possible autocrine ATB-centered regulatory loop that supports trastuzumab resistance and metastasis in BC [219].

Other individual studies revealed novel lncRNAs with oncogenic or tumor suppressor functions in BC drug-resistant tissues (Table 5). For instance, lncRNA-small nucleolar RNA host gene 14 (SNHG14) was shown to induce trastuzumab resistance via Bcl-2/Bax signaling modulation [220]. Similarly, Actin filament associated protein 1 antisense RNA1 (AFAP1-AS1)-enriched exosomes transferred anti-HER2 drugs resistance phenotype to the sensitive BC cells via AU-binding factor 1 (AUF1) regulation, which further enhanced ERBB2 translation [23]. Additionally, H19 was associated with the occurrence of trastuzumab resistance, as its experimental suppression resulted in increased therapeutic efficiency; however, the mechanisms exploited by H19 in BC targeted-therapy resistance remain to be further investigated [221]. Moreover, UCA1 acts as an oncogene in HER2-positive BC cells and sequestrates miR-18a leading to Yes-associated protein 1 (YAP1) overexpression and trastuzumab resistance [222]. Similarly, terminal differentiation-induced non-coding RNA (TINCR), present in elevated levels in the BC cells cytoplasm, may act as a sponge for miR-125b, upregulating HER-2 and Snail to induce resistance to the same antineoplastic agent in BC [223]. Increased expression of HOTAIR was shown to drive trastuzumab refractoriness in HER-2 positive BC and HOTAIR knockdown resulted in trastuzumab sensitization, and TGF-β, Snail, Vimentin, p-AKT, p-APK, and CyclinD1 suppression, respectively, E-cadherin, PTEN and P27 overexpression [224].

GAS5 is, in contrast, an essential sensitizer to anti-HER2 drug resistance in BC. GAS5 was found considerably downregulated in lapatinib resistant HER2-positive BC cells and tissues and was correlated with histological grading and advanced stage. Mechanically, GAS5 sponges miR-21 and increases the expression level of its target gene PTEN. In the same study, it was demonstrated that mTOR signaling activation might be responsible for GAS silencing and the occurrence of drug resistance in HER2-positive BC [225].

### 4.3. lncRNAs and BC Chemoresistance

lncRNAs have been identified to modulate multiple chemoresistance mechanisms in BC, bringing alluring perspectives regarding their use as diagnostic and prognostic tools in the clinical management of mammary carcinomas.

Several lncRNAs have been documented to act on components of apoptotic pathways to modulate chemoresistance in BC (Table 6). For instance, lncRNA P21-associated ncRNA DNA damage-activated (PANDA) was shown to promote doxorubicin resistance by binding it to the Nuclear Transcription Factor Y Subunit Alpha (NF-YA). Subsequently, the reduction of the levels of the pro-apoptotic genes Apoptotic protease activating factor 1 (APAF-1), Bumped kinase inhibitor (BKI), Fas Cell Surface Death Receptor (FAS), and Leucine-rich repeats and death domain-containing (LRDD) occurs [226]. lncRNA Adriamycin Resistance Associated (ARA) was also associated with the development of chemoresistance in BC. It was further shown that lncRNA ARA knockdown sensitized BC cells to anthracyclines, triggering apoptosis, G2/M arrest, and impaired cancer cell motility. Notably, lncRNA ARA silencing led to the upregulation of the pro-apoptotic protein Bcl-2 Associated X (BAX) and downregulation of the antiapoptotic B-cell lymphoma extra-large (Bcl-xL) protein, highlighting their roles as valuable target genes. Pathway analysis further elucidated that besides apoptosis, lncRNA ARA-target genes are enriched in multiple signaling pathways, such as MAPK and Peroxisome proliferator-activated receptor (PPAR) signaling, focal adhesion, purine, and pyrimidine metabolism, and cell cycle progression [227]. Likewise, the recently annotated LncRNA NONHSAT141924 was associated with therapeutic failure in BC. lncRNA NONHSAT141924 depletion restored paclitaxel sensitivity via modulating the phospho-cAMP-response element-binding protein (p-CREB)/Bcl-2 axis in BC [228]. Another study recognized the effect of upregulated lncRNA H19 in exacerbating BC chemoresistance. The experimental induction of lncRNA H19 was shown to attenuate paclitaxel-induced apoptosis in ERα-positive BC via epigenetic regulation of the key pro-apoptotic genes BCL2 Interacting Killer (BIK) and Phorbol-12-myristate-13-acetate-induced protein 1 (NOXA) in an EZH2-dependent manner [229].

Other lncRNAs have been documented to exert their regulatory functions on crucial players of DNA damage response and cell survival pathways, significantly impacting the therapeutic response in BC. For example, lncRNA BMP/OP-Responsive Gene (BORG) is an oncogenic lncRNA triggered in TNBC cells by the cellular stress exerted by chemotherapeutics. BORG expression is fueled by Nuclear factor-κB (NF-κB) through a novel BORG-mediated feed-forward signaling loop. It endows cells with a chemoresistant phenotype through Replication Protein A1 (RPA1) activation, an essential protein involved in DNA damage repair. Therapeutic targeting of BORG and its downstream modulators resulted in doxorubicin resensitization in TNBC [230]. Interestingly, LINC00968 was shown to boost the adriamycin’s therapeutic efficiency through WNT2 downregulation and the subsequent disruption of the Wnt2/β-catenin-associated signaling [231]. Similarly, the tumor suppressor lncRNA GAS5 was shown to target Wnt/β-catenin signaling and attenuate ABCB-1-mediated adriamycin resistance via miR-221-3p/Dickkopf-related protein 2 (DKK2) pathway regulation [232].

Evidence for the mechanistic link between lncRNAs and cell cycle dysfunction comes from a considerable number of studies. lncRNA Mitosis-Associated Long Intergenic Non-Coding RNA 1 (MA-linc1), involved in cell cycle control and M-phase exit, was overexpressed in paclitaxel-resistant BC cells. Its silencing increased the number of cells in the G2/M phase and enhanced paclitaxel-induced apoptosis, highlighting the crucial role of this lncRNA in proliferative function regulation [233]. UCA1, also part of the paclitaxel resistance signature in BC, was shown to act as a molecular sponge for miR-613, leading to elevated CDK12 levels [234]. Genome-wide profiling analysis of lncRNA expression revealed that 4030 lncRNAs and unannotated transcripts were upregulated, and 3708 were downregulated between anthracycline-resistant and sensitive MCF-7 cells. Among them, lncRNA NONHSAT02871 was found to regulate nearby CDK2 mRNA levels, thereby supporting cell cycle progression and chemoresistance in BC. Additionally, two other dysregulated lncRNAs, NONHSAT057282, and NONHSAG023333, were found to influence BC chemosusceptibility. It was further shown that these lncRNAs interacted with ELF1 and E2F1, respectively, to trans-regulate several chemoresistance-related genes such as Glutathione S-Transferase Pi 1 (GSTP1), BTG Anti-Proliferation Factor 3 (BTG3), Suppressor of Cytokine Signaling 3 (SOCS3), and breast cancer susceptibility gene (BRAC2) [235].

Other authors have highlighted the regulatory influences of lncRNAs on cancer stemness and EMT-related players. For instance, lncRNA nuclear paraspeckle assembly transcript 1 (NEAT1), highly upregulated in TNBC tissues, was shown to confer cisplatin chemoresistance via regulating CSCs biomarkers CD44, CD24, aldehyde dehydrogenase (ALDH), and SRY-related HMG-box 2 (SOX2) [22]. In addition to its involvement in DNA Damage Repair, lncRNA in non-homologous end joining (NHEJ) pathway 1 (LINP1) has been shown to act on EMT key regulators to support resistance to doxorubicin and 5-FU. The p53-regulated LINP1 has been associated with lower overall survival and a poor prognosis. Its siRNA-mediated knockdown has been associated with the conversion of BC cells to an epithelial state and the inhibition of chemoresistance [236]. Similarly, ZEB 1 antisense RNA 1 (ZEB1-AS1) depletion was proved to restore cisplatin sensitivity in BC by increasing miR-129-5p levels and negatively regulating its target gene ZEB1 [237]. Moreover, lncRNA ROR has been shown to render BC cells refractory to conventional therapies via N-cadherin and vimentin upregulation and E-cadherin downregulation in BC [238]. Additionally, it was observed that lncRNA ROR links EMT with ABC transporters in BC chemoresistance. This lncRNA modulates ABCB1 mRNA and protein levels in MDR-BC cells, and its inhibition resulted in decreased ABCB1-drug effuxing activity and enhanced chemosensitivity [239].

Several recent studies have highlighted various lncRNA signatures that may be used as biomarkers in monitoring the therapeutic response in BC. One such study was led by Wang, who identified 36 dysregulated lncRNAs that may serve as biomarkers for the response to neoadjuvant chemotherapy in BC, but further research regarding their biological functions has not yet been conducted [240]. Another study, led by Zeng et al., 2019, highlighted that elevated levels of lncRNAs AK291479 and BC032585 and low levels of lncRNA U79293 are indicators of pCR after neoadjuvant chemotherapy. Weighted gene co-expression network analysis and functional gene annotation revealed that these three molecules are involved in cell cycle regulation. To investigate the regulatory functions of BC032585 in this context, BC032585 was silenced, and this resulted in chemoresistance via MDR1 upregulation in BC [241]. We believe that further studies aiming at elucidating specific lncRNA expression signatures associated with various chemoresistance phenotypes will provide a more accurate picture of the magnitude of transcriptome changes induced by the chemotherapeutic stress in cancers, including BC.

## 5. ncRNAs and their Predictive Value in BC

Although the clinicopathological factors, such as tumor size, tumor grade, intrinsic subtype, and lymph node status are relevant to BC prognosis, they are unsatisfactory for predicting treatment outcomes in BC patients [246]. This has prompted the search for more dynamic biomarkers, such as miRNAs and lncRNAs, that can assist in predicting survival, metastasis, and patients’ prognosis following therapy. Their variable expression may distinguish between responsive and non-responsive groups, allowing the detection of resistance at an early stage [247].

Several studies have focused on ncRNAs involved in responsiveness to endocrine therapies. For example, Zhong and colleagues found that high levels of miR-30c independently predicted the clinical benefit of tamoxifen in ER-positive BC patients [248]. Conversely, Uhr et al. found that miR-7 is associated with poor PFS and OS in TAM-treated BC patients [249]. Other studies analyzed ncRNAs able to predict responsiveness to other therapies. The elevated miR-21 expression has been correlated with recurrence and poor OS in HER2-enriched-trastuzumab-treated patients [84]. High circulating levels of miR-375 and low levels of miR-122 were associated with an improved OS for stage II–III HER2-positive BC patients who underwent chemotherapy [250]. Some other studies suggested that miR-221, miR-222, and miR-155 are some of the most important indicators of poor prognosis in BC patients receiving chemotherapy; their increased expression is associated with lymph node metastasis, and tumor grade, which results in disease recurrence and patient death [251,252]. Some other ncRNAs with predictive value in BC are summarized in Table 7.

The majority of studies conducted so far assessed the predictive ability of a single ncRNA, which may be unsatisfactory for predicting complex biological events such as resistance or metastasis [253]. However, the current research has shifted towards the identification of ncRNA signatures incorporating multiple molecules that may be more relevant for the course of the disease. For instance, Wang et al. identified an 11-lncRNAs-based signature that could predict longer RSF in ER-positive BC patients treated with tamoxifen [204]. On the other hand, Tang et al. developed a lncRNA signature indicative of recurrence in ER-positive BC patients receiving endocrine therapy [254]. Jiang and colleagues also discovered an mRNA-lncRNA signature able to subdivide TNBC patients into groups with high or low risk of relapse. Additionally, the signature could predict response to taxane-based chemotherapy in these patients [255]. Although research concerning ncRNAs as predictive biomarkers for BC is still in its infancy, someday, they will have their place in the precision medicine initiative.

## 6. Discussion

Breast cancer is the most commonly diagnosed malignancy in women and has become the second leading cause of cancer-related deaths among females globally. It is a heterogeneous disease with variable molecular underpinnings, leading to considerable differences in prognosis, propensity to metastasize, therapeutic responses, and long-term patient survival. Although the use of systemic therapies (targeted therapy, endocrine therapy, and chemotherapy) has greatly improved pathological complete response and overall survival, a robust body of evidence has shown that not all BC patients are sensitive to conventional therapies, due to the innate and/or acquired drug resistance [33]. To overcome the challenges posed by the formidable heterogeneity of BC, at the moment, the efforts of the scientific community are oriented towards the identification of novel biomarkers with diagnostic, predictive, and prognostic power in this clinical setting. Recently, non-coding RNAs (ncRNAs), especially microRNAs (miRNAs) and long non-coding RNAs (lncRNAs) emerged as key regulators in BC drug resistance via modulating its canonical pathways at epigenetic, transcriptional, and post-transcriptional levels. Due to their increased stability in body fluids and their ubiquitous involvement in BC intracellular and intercellular signaling, ncRNAs are regarded as promising biomarkers in BC management and are expected to open new directions of research in precision medicine [35,47].

**Figure 3:**
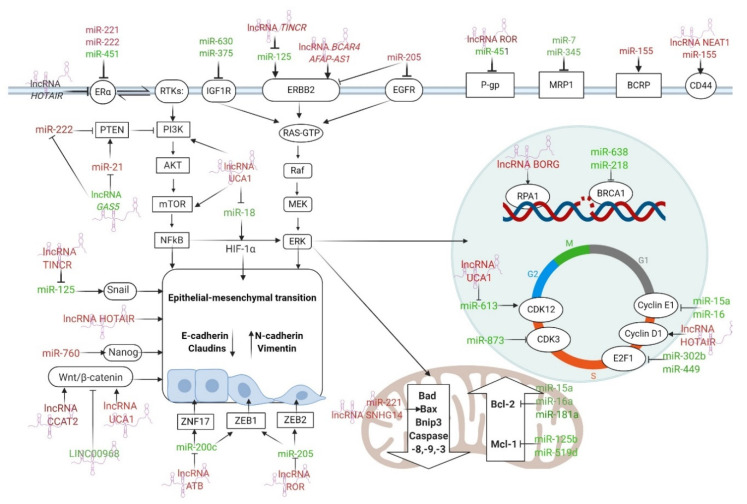
The most important ncRNAs that regulate ER and HER2 receptors, drug efflux, apoptosis, DDR, cell cycle arrest, EMT, and CSCs features in BC drug resistance. The arrows indicate the stimulation of the signaling pathway, while the T-bars indicate the inhibition of the signaling pathway. ncRNAs with tumor suppressor functions are stained green, while oncogenic ncRNAs are stained red. Figure created with Biorender.com.

Here, we reviewed the literature on the involvement of miRNAs and lncRNAs in the mechanisms that render BC resistant to medication and identified several ncRNAs that can serve as potential biomarkers of response to systemic therapies (endocrine therapy, targeted therapies, and chemotherapy) in BC. These molecules have been shown to amplify or weaken BC drug resistance via regulating apoptosis/cell survival pathways, drug targets, epithelial–mesenchymal transition (EMT), cancer stemness, cell cycle progression, drug efflux mechanisms, as well as many other pathways (Figure 3). The use of anti-miRNAs and miRNA mimics to deplete oncogenic miRNAs and simulate endogenous tumor suppressor miRNAs, is documented to reverse BC drug resistance in different experimental systems successfully. Therefore, the administration of certain RNA-based formulations in conjunction with systemic therapies is a promising strategy for combating the burden of drug resistance in BC.

**Figure 4:**
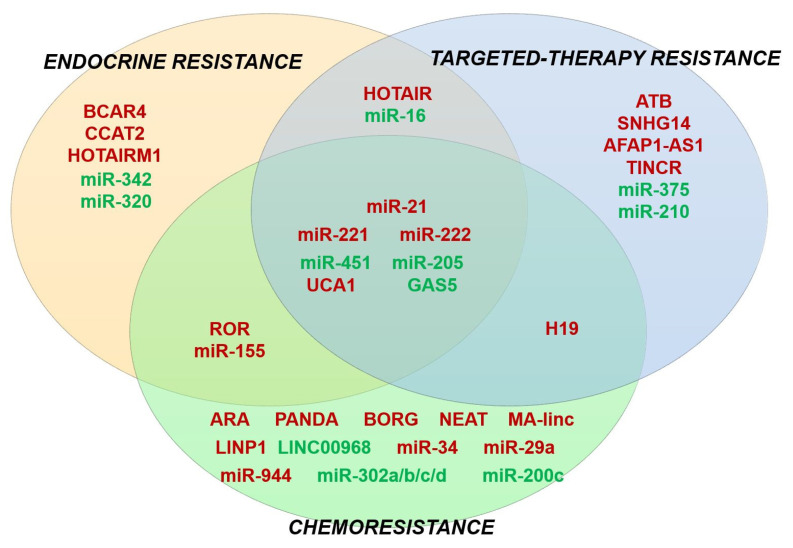
Venn diagram showing an overlapping role of several miRNAs and lncRNAs in multiple drug-resistant phenotypes in BC. ncRNAs with tumor suppressor functions are stained green, while oncogenic ncRNAs are stained red.

However, it is challenging to choose ncRNAs with the highest therapeutic potential among a plethora of potential candidates. Our attention, as well as that of the scientific community, is focused on the ncRNAs that regulate multiple drug resistance phenotypes in BC and other cancers. Such examples can be found in Figure 4, in the regions where different sets of drug resistance associated-ncRNAs overlap. Interestingly, besides its involvement in BC drug resistance, lncRNA UCA1 was shown to promote chemoresistance against various agents in different cancers, such as prostate cancer (docetaxel) [266], ovarian cancer (cisplatin) [263], colorectal cancer (5-FU) [267], chronic myeloid leukemia (imatinib) [268], bladder cancer (cisplatin, gemcitabine) [269], and gastric cancer (MDR) [270]. Additionally, overexpressed miR-221 was shown to enhance cisplatin resistance in osteosarcoma via Protein phosphatase 2 (PP2A) targeting [271], but also rendered pancreatic cancer cells refractory to 5-FU through regulating the gene encoding for Retinoblastoma Protein 1 (RB1) [272]. lncRNA GAS5 was also found to enhance the therapeutic efficiency in various cancers, such as non-small cell lung cancer (NSCLC) [273], pancreatic cancer [274], and prostate cancer [275], thereby acting as a valuable tumoral suppressor. However, the enthusiasm generated by the therapeutic power of these molecules is tempered by several challenges that need to be overcome as soon as possible. These include the need for validation in the clinical setting, standardization of current methodologies for ncRNA isolation and quantification, and the investigation of the in vivo effects of artificial miRNAs and lncRNA mimics in complex interactions such as competitive endogenous RNAs (ceRNAs) [276,277,278].

## 7. Conclusions

Undoubtedly, BC drug resistance and ncRNAs are two hot topics of our modern times; therefore, we consider that further characterization of these ncRNAs in relationship with their genomic functions and impact on chromatin architecture is likely to provide novel biomarkers for validation in clinical cohorts, which may be ultimately for patient risk stratification and optimization of therapeutic protocols in BC.

## Figures and Tables

**Table 1:** miRNAs regulating endocrine resistance in BC ^1^; (u) upregulated; (d) downregulated; (+) increase; (−) reduction.

ncRNAs	Drug	Status	Impact on Drug Resistance	Target(s)	Mechanism(s)	Experimental System	References
miR-342	Tamoxifen	d	+	BMP7, GEMIN4	Apoptosis evasion	MCF-7	[78]
miR-320	Tamoxifen	d	+	ARPP-19, ERRγ	Cell proliferation	MCF-7, T47D	[79]
miR-451a	Tamoxifen	u	−	14-3-3ζ, ERα	Erα signaling and cell survival	MCF-7	[80]
miR-15a/16	Tamoxifen	u	-	Cyclin E1, Bcl-2	Cell cycle regulation, apoptosis evasion	MCF-7	[81]
miR-21	Tamoxifen fulvestrant	u	+	PTEN	PI3K/Akt activation, autophagy	MCF-7	[82]
miR-155-5p	Tamoxifen	u	+	SOCS6	STAT3 pathway	MCF-7, SKBR3	[87]
miR-221/222	Tamoxifen fulvestrant	u	+	p27, ERα	Erα signaling, cell cycle regulation, EMT	MCF-7, BT474	[55,88]
miR-200	Tamoxifen	d	+	MYB	EMT	MCF-7	[89]
miR-873	Tamoxifen	d	+	CDK3	Erα signaling and cell cycle progression	MCF-7	[90]
miR-10b	Tamoxifen	u	+	HDAC	TGF-β1-induced EMT	MCF-7, T47D	[91]
miR-148a miR152	Tamoxifen	u	−	ALCAM	Tumor growth and survival	MCF-7	[92]
miR-27b-3p	Tamoxifen	d	+	NR5A2/CREB1	Cell proliferation	MCF-7, T47D	[93]
miR-27b	Tamoxifen	d	+	HMGB3	EMT	MCF-7	[94]
miR-214	Tamoxifen fulvestrant	u	−	UCP-2	Autophagy	MCF-7	[95]

^1^ p27—cyclin-dependent kinase inhibitor 1B; 14-3-3ζ—14-3-3 protein zeta; Bcl-2—B-cell lymphoma 2; MYB-MYB proto-oncogene; CDK3—Cyclin Dependent Kinase 3; SOCS6—Suppressor Of Cytokine Signaling 6; HDAC—histone deacetylase; ALCAM—activated leukocyte cell adhesion molecule; NR5A2/CREB1—Nuclear Receptor Subfamily 5 Group A Member 2/CAMP responsive element binding protein 1; HMGB3—High Mobility Group Box 3; UCP-2—Uncoupling Protein 2; BMP7—bone morphogenetic protein 7; GEMIN4—Gem Nuclear Organelle Associated Protein 4; ARPP-19—CAMP Regulated Phosphoprotein 19; ERRγ—estrogen-related receptor gamma.

**Table 2:** miRNAs involved in targeted therapy resistance in BC ^1^; (u) upregulated; (d) downregulated; (+) increase; (−) reduction.

ncRNAs	Drug	Status	Impact on Drug Resistance	Target(s)	Mechanism(s)	Experimental System	References
miR-205-5p	Trastuzumab	u	+	ERBB2, EGFR	Cell survival	BCSCs	[24]
miR-205	Trastuzumab	u	−	NA	Akt impairment	SKBR3	[26]
mir-375	Trastuzumab	u	−	IGF1R	Cell survival	SKBR3	[99]
miR-630	Lapatinib neratinib afatinib	u	−	IGF1R	Cell survival	SKBR3, HCC1954	[100]
miR-210	Trastuzumab	u	−	E2F3, RAD52	DNA damage repair and cell proliferation	BT474, patient serum	[101,102]
miR-21	Trastuzumab	u	+	PTEN, PDCD4	PI3K/Akt activation, EMT and inflammatory signals	SKBR3, BC patient tissues	[103]
miR-221	Trastuzumab	u	+	PTEN	PI3K/Akt/mTOR pathway	SKBR3	[104]
miR-16	Trastuzumab lapatinib	u	−	CCNJ, FUBP1	Cell proliferation and survival	BT474, SKBR3, HCC-1569, MDA-MB-453	[105]
miR-567	Trastuzumab	d	+	ATG5	Autophagy	SKBR-3, BT474	[106]
miR-200c	Trastuzumab	u	−	ZNF217, ZEB1	TGF-β signaling-induced EMT	SKBR3	[107]
miR-7	Trastuzumab	u	-	EGFR, Src kinase	ERBB2-driven proliferation	MCF-7	[108]

^1^ PDCD4—Programmed Cell Death 4; RAD52—RAD52 Homolog; ZNF217—Zinc Finger Protein 217; ZEB1—Zinc Finger E-Box Binding Homeobox 1; Src kinase—proto-oncogene tyrosine-protein kinase Src; ATG5—autophagy related 5; TGF-β—transforming growth factor beta.

**Table 3:** miRNAs regulating BC chemoresistance ^1^; (u) upregulated; (d) downregulated; (+) increase; (−) reduction.

ncRNA(s)	Drug	Status	Impact on Drug Resistance	Target(s)	Mechanism(s)	Experimental System	References
miR-302a-3p miR-302b-3p miR-302c-3p miR-302d-3p	Doxorubicin	u u u u	− − − −	MEKK1/P-gp	Drug efflux	MCF-7	[25]
miR-451	Doxorubicin	d	+	P-gp (ABCB1/MDR-1)	Drug efflux	MCF7	[111]
	Paclitaxel	u	−	YWHAZ	Cell cycle control, apoptosis and invasion	MCF-7, SKBR3	[142]
miR-298	Doxorubicin	u	−	P-gp	Drug efflux	MDA-MB-231	[143]
miR-326	Doxorubicin VP-16	d	+	ABCC1 (MRP-1)	Drug efflux	MCF-7	[144]
miR-320a	Doxorubicin paclitaxel	d	+	TRPC5, NFATC3, ETS1	P-gp-mediated drug efflux	MCF-7	[145]
miR-145-5p	Doxorubicin	u	−	ABCC1	Drug efflux	MCF-7	[146]
miR-128	Doxorubicin	d	+	Bmi1, ABCC5	Drug efflux, stemness	SK-3RD and MCF-7 mammosphere	[147]
miR-25a	Adriamycin	u	+	Unc51	ABCG2 regulation	MCF-7	[126]
miR-487a	Mitoxantrone	d	+	ABCG2 (BCRP)	Drug efflux	MCF-7	[148]
miR-449	Doxorubicin	u	−	E2F1, E2F3, CDK2	Cell cycle regulation	MDA-MB-231	[113]
miR-1268b	Doxorubicin	u	−	ERBB2	PI3K/Akt pathway regulation	MCF-7	[149]
miR-222 miR-29a	Doxorubicin docetaxel	u u	+ +	PTEN	PI3K/Akt pathway activation	MCF-7	[116]
miR-34a	MDR	d	+	Bcl-2, CCND1, Notch	Survival pathways, cell cycle progression	MCF-7, MDA-MB-231	[54,150,151]
		d	+	HDAC1, HDAC7	Cell survival and autophagy	MCF-7, MDA-MB-231	[152]
miR-29a	Doxorubicin	u	+	PTEN	PI3K/Akt/GSK3β signaling regulation	MCF-7	[153]
miR-31	Doxorubicin	u	−	PKCepsilon/Bcl2 axis	Cell survival	MCF10A, MDA-MB-231	[124]
miR-222	Doxorubicin	u	+	Bim	Apoptosis/survival pathways	MCF-7, patients’ serum	[154]
	Doxorubicin	u	+	PTEN/Akt/p27 ^kip1^	Survival pathway	MCF-7	[155]
miR-708	Doxorubicin	d	+	ZEB1, CDH2, vimentin	EMT	MCF-7, MDA-MB-468	[156]
miR-218	Doxorubicin taxol	u	−	Survivin	Apoptosis	MCF-7, Cal51, mouse tumor xenografts	[57]
miR-141, miR-200c, miR-31 let-7e, miR-576-3p, miR-125b-1, miR-370, miR-145, miR-765, miR-760	Doxorubicin	u d	+ +	Genes involved in MAPK signaling pathway, regulation of the actin cytoskeleton, cytokine–cytokine receptor interaction		MCF-7	[157]
miR-302b	Cisplatin	u	−	E2F1, ATM signaling	Cell cycle progression, DNA damage repair	MDA-MB-231	[112]
miR-221	Cisplatin	d	−	Bim-Bax/Bak	Apoptosis	MDA-MB-231	[122]
miR-30c	Doxorubicin paclitaxel	d	+	TWF1/IL-11	EMT	T47D, MCF-7, MDA-MB-231, BT-20, HCC-70, HCC-38	[131]
miR-3646	Docetaxel	u	+	GSK-3β/β-catenin	Cell survival and proliferation	MDA-MB-231, MCF-7	[158]
miR-224-3p	MDR	d	+	FUT4	Cell growth and survival	T47D	[159]
miR-193b	Doxorubicin	d	+	MCL-1	Apoptosis	MCF-7	[160]
miR-21-5p	Docetaxel	u	+	PTEN	Cell survival	MCF-7	[161]
miR-133a	Doxorubicin	d	+	UCP-2	Cell metabolism alteration	MCF-7, mouse xenograft models	[162]
miR-200a	Cisplatin gemcitabine paclitaxel	u	+	TP53INP1, YAP1	EMT	ZR-75-30, MDA-MB-231	[163]
miR-149	Doxorubicin	d	+	NDST1	Tumor growth and angiogenesis	MCF-7	[164]
miR-141	Docetaxel	u	+	EIF4E	Apoptosis	MCF-7, MDA-MB-231	[165]
miR-125b	Doxorubicin	u	−	Mcl-1	Apoptosis	MCF-7	[166]
	Paclitaxel	u	−	Sema4C	EMT	MCF7, SKBR3	[167]
	Paclitaxel	u	+	Bak1	Apoptosis	MDA-MB-435, MDA-MB-436, SKBR3	[168]
	Doxorubicin	u	−	HAX1	Apoptosis	MCF-7	[169]
miR-129-3p	Docetaxel	u	+	CP110	Cell cycle control and apoptosis	MDA-MB-231, MCF-7, nude xenograft model	[170]
miR-542-3p	Paclitaxel	u	−	Survivin	Apoptosis	SKBR3, BT474, MDA-MB-453, HCC1954	[118]
miR-25-3p	Epirubicin	d	−	ULK1	Autophagy	MCF-7	[126]
miR-139-5p	Docetaxel	d	+	Notch-1	Cell cycle control and survival, invasion	MCF-7	[171]
miR-760	Doxorubicin	d	+	Nanog	EMT	MCF-7	[172]
miR-484	Gemcitabine	u	−	CDA	Proliferation and cell cycle control	MDA-MB-231	[173]
miR-489	Doxorubicin	u	−	ULK1, LAPTM4B	Autophagy and cell viability	T47D, MDA-MB-231	[28]
	5-FU	u	−	XIAP	Survival pathways	T47D, SKBR3	[174]
	Doxorubicin	u	−	SPIN1 Smad3	PI3K/Akt modulation, EMT	MCF-7	[175] [176]
miR-100	Paclitaxel	u	−	mTOR	Apoptosis, cell cycle control	MCF-7, MDA-MB-231	[117]
miR-663a	Doxorubicin	u	+	HSPG2	Antiapoptotic response	MDA-MB-231	[177]
miR-200c	Doxorubicin	u	−	TrkB, Bmi1	Cell survival and EMT	BT474	[178]
	Carboplatin doxycycline	u	−	EZH2, Bmi1	EMT	p53-null claudin-low tumor model	[133,134]
miR-105 miR-93-3p	Cisplatin	u	+	SFRP1/Wnt/β-catenin	Stemness and EMT induction	HCC70, MDA-MB-231, BT-549, HCC1937	[179]
miR-24	Cisplatin	u	+	BimL, FIH1-HIF1α	Cell survival and EMT	T47D, MDA-MB-231, MCF-7, BT-549	[180]
miR-20a	MDR	u	−	MAPK1/c-Myc/P-gp	Survival pathways, drug efflux	BCap37, Bads-200, Bats-72	[181]
miR-424(322) miR-503	Paclitaxel	d	+	BCL-2, IGF1R	Apoptosis/survival pathways	Mouse model	[121]
miR-638	Cisplatin	u	-	BRCA1	DNA damage repair	MCF-7, MDA-MB-231, T47D, Hs 578T	[114]
miR-519d	Cisplatin	u	−	MCL-1	Apoptosis	T47D, T47D-CSCs	[129]
miR-181a	Doxorubicin	d	+	Bcl-2	Apoptosis	MCF-7	[120]
	Mitoxantrone	u	−	ABCG2 (BCRP)	Drug efflux	MCF-7	[182]
	Doxorubicin	u	+	Bax	Apoptosis	MDA-MB-231	[183]
miR-27b-3p	MDR	d	+	CBLB/GRB2	PI3K/Akt and MAPK/Erk signaling pathways regulation	Bcap37, Bads-200, MCF-7, MDA-MB-231	[59]
miR-520h	Paclitaxel	u	+	DAPK2	Apoptosis evasion		[184]
miR-18a	Paclitaxel	u	+	Dicer	Apoptosis evasion	MDA-MB-231	[185]
miR-101	Paclitaxel	u	+	MCL-1	Apoptosis evasion	MDA-MB-435	[186]
miR-345 miR-7	Cisplatin	d d	+ +	MRP-1	Drug efflux	MCF-7	[53]
miR-944	Cisplatin	d	−	BNIP-3	Apoptosis	MCF-7	[123]
miR-199a-3p	Cisplatin	u	−	TFAM	Apoptosis	MDA-MB-231	[187]
miR-218	Cisplatin	u	−	BRCA1	DNA damage repair	MCF-7	[115]
miR-21	Gemcitabine	u	+	PTEN/Akt pathway	EMT	MDA-MB-231	[83]
miR-205	MDR	u	−	VEGFA, FGF2	Cell growth and angiogenesis	MCF-7, Cal51	[27]
miR-137	MDR	u	+	FSTL1	Wnt/β-catenin related cellular stemness	MDA-MB-231	[29]
miR-140	MDR	u	−	Wnt/β-catenin signaling	Cellular stemness	MCF-7, MDA-MB-231	[127]
miR-155	Doxorubicinol	u	+	CD44, CD90, ABCG2	Cellular stemness and drug efflux	MDA-MB-231	[128]

^1^ YWHAZ—14-3-3 protein zeta/delta; CCND1—Cyclin D1; HDAC—Histone Deacetylase 1; Bim—Bcl-2-like protein 11; ATM—ataxia telangiectasia mutated; TWF1—Twinfilin Actin Binding Protein 1; IL11—interleukin 11; GSK-3β—glycogen synthase kinase 3 beta; FUT4—Fucosyltransferase 4; UCP2—Uncoupling Protein 2; TP53INP1—Tumor Protein P53 Inducible Nuclear Protein 1; YAP1—Yes Associated Protein 1; CDH2—Cadherin 2; EIF4E—Eukaryotic Translation Initiation Factor 4E; NDST1—N-Deacetylase And N-Sulfotransferase 1; CP110—centriolar coiled coil protein 110; Sema4C—Semaphorin 4C; Bak1—Bcl2 antagonist/killer 1; Hax1—HCLS1-associated protein X-1; TRPC5—Transient Receptor Potential Cation Channel Subfamily C Member 5; NFATC3—Nuclear Factor Of Activated T Cells 3; ETS1—ETS Proto-Oncogene 1; ULK1/LAPTM4B—Unc-51-like kinase 1/Lysosomal Protein Transmembrane 4 Beta; CDA—cytidine deaminase; XIAP—X-Linked Inhibitor Of Apoptosis; HSPG2—Heparan Sulfate Proteoglycan 2; TrkB—tropomyosin receptor kinase B; Bmi-1—BMI1 Proto-Oncogene/Polycomb Ring Finger protein; FIH1—factor inhibiting hypoxia-inducible factor 1 alpha; HIF1α—hypoxia-inducible factor-1 alpha; TFAM—Transcription Factor A, Mitochondrial; FSTL1—follistatin-related protein 1.

**Table 4:** Oncogenic and tumor suppressor lncRNAs associated with BC endocrine resistance ^1^; (u) upregulated; (d) downregulated; (+) increase; (−) reduction.

ncRNA(s)	Drug	Status	Impact on Drug Resistance	Target(s)	Mechanism(s)	Experimental System	References
ROR	Tamoxifen	u	+	miR-205/ZEB1, ZEB2	TGF-β-induced EMT	MCF-7, MDA-MB-231	[202]
HOTAIR	Tamoxifen	u	+	ER signaling	Cell proliferation	MCF-7	[205]
BCAR4	Tamoxifen	u	+	ERBB2 and ERBB3 signaling	Cell survival and proliferation	ZR-75-1	[207]
CCAT2	Tamoxifen	u	+	Wnt/β-catenin pathway	Cell survival and viability	MCF-7, T47D	[209,211]
UCA1	Tamoxifen	u	+	EZH2/p21	Akt/mTOR signaling	MCF-7 T47D	[213,214]
		u	+	Wnt/β-catenin pathway	Cell survival and EMT	MCF-7, T47D	[215]
		u	+	miR-18a/HIF1α axis	Cell cycle regulation	BT474	[216]
GAS5	Tamoxifen	d	+	miR-222/PTEN	PI3K/Akt/mTOR signaling	MCF-7	[218]

^1^ ERBB3—Erb-B2 Receptor Tyrosine Kinase 3; EZH2—Enhancer of Zeste 2 Polycomb Repressive Complex 2 Subunit; p21—cyclin dependent kinase inhibitor 1A; TGF-β—transforming growth factor beta.

**Table 5:** lncRNAs in anti-HER2 drug resistance ^1^; (u) upregulated; (d) downregulated; (+) increase; (−) reduction.

ncRNA(s)	Drug	Status	Impact on Drug Resistance	Target(s)	Mechanism(s)	Experimental System	References
AFAP-AS1	Trastuzumab	u	+	AUF1/ERBB2	Cell survival	SKBR3, BT474	[23]
ATB	Trastuzumab	u	+	miR-200c/ZEB1, ZNF-217	EMT	SKBR3, BC patient tissues	[56]
SNHG14	Trastuzumab	u	+	Bcl2/Bax	Apoptosis	SKBR3, BT474	[220]
H19	Trastuzumab	u	+	NA	NA	BC patients	[221]
UCA1	Trastuzumab	u	+	miR-18a/YAP1	Cell viability	SKBR3	[222]
TINCR	Trastuzumab	u	+	miR-125b/HER2, Snail	EMT	SKBR3, BT474	[223]
HOTAIR	Trastuzumab	u	+	TGF-β, Snail, Vimentin, p-AKT, CyclinD1, E-cadherin, PTEN, P27	Cell cycle and proliferation, EMT	SKBR3	[224]
GAS5	Trastuzumab, lapatinib	u	−	miR-21/PTEN	AKT/mTOR modulation	SKBR3	[225]

^1^ AUF1—AU-rich element RNA-binding factor 1; YAP1—Yes Associated Protein 1; TGF-β—transforming growth factor beta.

**Table 6:** Several lncRNAs involved in regulating BC drug resistance to anthracyclines, taxanes, platinum compounds, and antimetabolites ^1^; (u) upregulated; (d) downregulated; (+) increase; (−) reduction.

ncRNA(s)	Drug	Status	Impact on Drug Resistance	Target (s)	Mechanism(s)	Experimental System	References
NEAT	Cisplatin taxol	u	+	CD44, CD24, SOX2	Cellular stemness	MDA-MB-231	[22]
H19	Antracyclines	u	+	CUL4A-ABCB1/MDR1	Drug efflux	MCF-7	[58]
	Paclitaxel	u	+	BIK, NOXA	Apoptosis	MCF-7, ZR-75-1	[229]
PANDA	Doxorubicin	u	+	NF-YA/APAF-1, BKI, FAS and LRDD	Apoptosis	Primary breast tumors	[226]
ARA	Doxorubicin	u	+	NA	MAPK and PPAR signaling, metabolic signaling pathways, cell cycle and focal adhesion	MCF-7	[227]
NONHSAT141924	Paclitaxel	u	+	p-CREB/Bcl2	Apoptosis	MCF-7	[228]
BORG	Doxorubicin	u	+	RPA-1	Cell survival and DNA damage repair	SKBR3, BT474	[230]
LINC00968	Adriamycin	u	−	Wnt2	Wnt/β-catenin survival pathway	MCF-7, KPL-4	[231]
GAS5	Adriamycin	d	+	miR-221/DKK2 and Wnt/β-catenin survival	Drug efflux	MCF-7	[232]
MA-linc1	Paclitaxel	u	+	Purα	Cell cycle regulation	U2OS	[233]
UCA1	Paclitaxel	u	+	miR-613/CDK12	Cell viability and apoptosis	MCF-7	[234]
NONHSAT028712 NONHSAT057282 NONHSAG023333	Doxorubicin	u u u	+ + −	CDK2 ELF1, E2F1, SOCS3, BRAC2	Cell cycle regulation and survival	MCF-7	[235]
LINP1	5-FU doxorubicin	u u	+ +	N/E-cadherin, vimentin Ku80	EMT DNA Damage Repair	MDA-MB-231	[236] [242]
ZEB1-AS1	Cisplatin	u	+	miR-129-5p/ZEB1	EMT	MCF-7	[237]
ROR	5-FU paclitaxel	u	+	N/E-cadherin, vimentin	EMT	BC tissues and lines	[238]
	MDR	u	+	ABCB1	Drug efflux	MDA-MB-231, Sum159PT	[239]
Linc00152	Doxorubicin	u	+	N-cadherin, E-cadherin, vimentin	EMT	MDA-MB-231, MCF-7	[243]
AX747207	Doxorubicin	d	+	RUNX3	PI3K/Akt, Hippo and ErbB oncogenic pathways	MCF-7	[244]
PRLB	5-FU	u	+	miR-4766-5p/SIRT1	Tumor growth and metastasis	MDA-MB-231/468	[245]

^1^ BIK—BCL2 Interacting Killer; NOXA—phorbol-12-myristate-13-acetate-induced protein 1; CUL4A—Cullin-4A; MDR1—multi-drug resistance-1/P-glycoprotein; NA—not available; CDK2—Cyclin Dependent Kinase 2; ELF1—E74-like factor 1; E2F1—E2F Transcription Factor 1; SOCS3—Suppressor of Cytokine Signaling 3; RPA-1—Replication Protein A1; RUNX3—RUNX Family Transcription Factor 3; SIRT1—Sirtuin 1; NF-YA—Nuclear Transcription Factor Y Subunit Alpha; APAF-1—apoptotic protease activating factor-1; BKI—bumped kinase inhibitor; FAS—Fas Cell Surface Death Receptor; LRDD—Leucine Rich Repeats And Death Domain Containing Protein; DKK2—Dickkopf WNT Signaling Pathway Inhibitor 2; Purα—purine-rich binding protein-α; ZEB1—Zinc Finger E-Box Binding Homeobox 1; Wnt2—Wnt Family Member 2; PPAR—peroxisome proliferator-activated receptor.

**Table 7:** Compendium of ncRNAs associated with the clinical outcome in BC ^1^; (↑)–increased;(↓)–decreased.

Clinical Outcome	ncRNA(s)	Biological Samples	Association	Reference(s)
Survival	↑miR-4653-3p	FFPE BC tissues	Increased DSF in ER-positive BC patients receiving TAM adjuvant therapy	[256]
	↑miR-30c-5p, ↑miR-↑30b-5p, ↓miR-182-5p, ↑miR-200b-3p	Fresh frozen BC tissues	Improved ERFS in endocrine therapy-treated BC patients	[257]
	↑miR-204	FFPE tissues	Improved DSF and OS in patients treated with chemotherapy; inverse correlation with TNM stage and metastasis	[258]
	↑miR-375	BC tissues and serum	Improved OS for stage II–III HER2-positive BC patients who underwent chemotherapy	[250]
	↑miR-30c	BC tissues	Response to TAM and longer PFS in ER-positive BC patients	[248]
	↑PINK1.AS, ↑KLF3.AS1, ↑LINC00339, ↑LINC00472, ↑RP11.351I21.11, ↑PKD1P6.NPIPP1, ↑PDCD4.AS1, ↑RP11.69E11.4, ↓RP11.259N19.1, ↓KB.1460A1.5, ↓PP14571	BC tissues	Longer RSF in ER positive BC patients treated with tamoxifen	[204]
Relapse	↑miR-7	BC cell lines and fresh frozen tissues	Poor PFS and post-relapse OS in TAM-treated BC patients	[249]
	↑miR-210	BC cell tissues; BC cells an TEMs in coculture	Reduced DSF and OS in tamoxifen treated ER-positive BC patients	[259,260,261]
	↑miR-21	Frozen tissues	Poor patient outcome in trastuzumab-treated patients	[84]
	↑miR-454	FFPE BC tissues	Worse DFS in anthracycline-treated TNBC patients	[262]
	↑miR-221/222	FFPE and frozen BC tissues, cell lines	Poor prognosis related to lymph node metastasis in BC patients undergoing chemotherapy	[251,263]
	↑miR-95-3p	FFPE BC tissues	Decreased OS and RFS in patients treated with anthracycline-based chemotherapy	[264]
	↑miR-222, ↑miR-29a, ↑miR-140, ↑miR-574, ↑miR-6780b, ↑miR-7107, ↑miR-744	FFPE BC tissues	Associated with decreased PFS and OS in chemotherapy-treated BC patients	[263]
	↑miR-125, ↑miR-21	Serum	Decreased DSF in patients receiving neoadjuvant chemotherapy	[265]
	↑miR-122	BC tissues and serum	Predicts metastatic recurrence in stage II–III HER2-positive BC patients who underwent chemotherapy	[250]
	↑miR-155	BC tissues	Associated with tumor grade and lymph node metastasis in paclitaxel-resistant BC patients	[252]
	↑HOTAIR	BC tissues	Metastatic disease and poor OS	[20]
	↑HIF1A-AS2, ↑AK124454	BC tissues	Poor outcome in paclitaxel-resistant TNBC patients	[255]
	↑FAM224A, ↑LINC00987, ↑MCM3AP-AS1, ↑RP11–351I21.11, ↑SNHG17, ↓CTA-228A9.4, ↓EGOT, ↓ HAND2-AS1	BC tissues	Poor OS in ER-positive patients receiving endocrine therapy	[254]

^1^ Endocrine resistance-free survival (ERFS); OS—overall survival; DFS—disease-free survival; PFS—progression-free survival; FFPE—formalin-fixed paraffin-embedded; TNBC—triple-negative breast cancer.
